# Influenza A (H1N1) pdm09 virus infection in a patient with incomplete Kawasaki disease: A case report

**DOI:** 10.1097/MD.0000000000015009

**Published:** 2019-04-12

**Authors:** Jun Wang, Fang Sun, Hui-Ling Deng, Rui-Qing Liu

**Affiliations:** a Department Second of Infectious Diseases; b Department of Respiratory, Xi’an Children's Hospital, Xi’an, Shaanxi Province, China.

**Keywords:** influenza A (H1N1) pdm09 virus, Kawasaki disease, pathogen infection

## Abstract

**Rationale:**

Kawasaki disease (KD) is a vasculitic illness of childhood associated with coronary artery dilatation, coronary artery aneurysm, arrhythmia, sudden death, and other serious cardiovascular diseases. Up to date, the etiology of KD remains unclear; however, epidemiological characteristics indicate that it may be related to as-yet-undefined pathogen infection.

**Patient concerns:**

A 19-month-old boy had a fever of unknown origin at 38°C for 9 days without rash, runny nose and cough.

**Diagnosis:**

The boy was diagnosed with incomplete KD (IKD) coincident with influenza A (H1N1) pdm09 virus.

**Interventions:**

He was received treatments including human immunoglobulin (2 g/kg), aspirin (30∼50 mg/kg.d), and dipyridamole (3∼5 mg/kg.d).

**Outcomes:**

After 24 hours of human immunoglobulin infusion, his body temperature returned normal. After hospitalization for 6 days, his symptoms disappeared and discharged from the hospital.

**Lessons:**

More attention should be paid to the correlation between KD and pathogen infection, especially the new influenza virus H1N1. The potential mechanism underlying viral infection-mediated KD is worthy of further investigation, which may provide scientific evidence for the pathogenesis of KD.

## 1 Introduction

Kawasaki disease (KD) is a vasculitic illness of childhood associated with coronary artery dilatation, coronary artery aneurysm, arrhythmia, sudden death, and other serious cardiovascular diseases. KD has become one of the most common causes of acquired heart disease in children.^[1]^ Since Kawasaki, a Japanese doctor, first described the disease in the late 1960s, there have been successive reports all over the world, and the incidence of the disease has risen. In clinical practice, many patients have incomplete (atypical) KD (IKD), as they do not fulfill the diagnostic criteria of KD. IKD accounts for about 16.1% of children with KD.^[2]^ Up to date, the etiology of KD remains unclear, however, epidemiological characteristics indicate that it may be related to as-yet-undefined pathogen infection. Influenza A (H1N1) pdm09 virus is a novel strain that emerged in 2009 and is highly infectious and transmissible, but the pathogenicity is mild, the lethal rate is low. Most of the infected children had a mild self-limiting viral illness, and only a few developed severe diseases. This article describes a case of IKD coincident with influenza A (H1N1) pdm09 virus, suggesting that influenza infection may be a potential cause of KD.

## 2 Case report

In February 2018, a previously healthy 19-month-old boy was admitted to our hospital because of febrile illness. Nine days previously he had a fever of unknown origin at 38°C without rash, runny nose and cough. Before admission, he received 4 days’ treatment of pediatric paracetamol, artificial cow-bezoar, ibuprofen suspension, and cefatriaxone, however, after the treatments; he still had fever up to 39.5°C. He developed paroxysmal barking cough at 1 day before admission. On admission, he was found with hoarse voice, mild breathing dyspnea in quiet, no irritability and cyanosis, throat swabs virus test showed “influenza A virus antigen positive”.

On examination, he was alert and upset. He febrile to 38°C had a pulse rate of 138/min, respiratory rate of 38/minute, blood pressure of 85/52 mmHg, and blood oxygen saturation of 94% in room air. His hips were slightly flush, and his finger-end (toe-end) was red but not swollen. No rash in the body, no tenderness, no hyperemia in his bulbar conjunctiva, no chap, and no strawberry-like tongue was noted. There was weak positive tri-retraction sign, and double lung had a laryngeal conduction sound. The heart sounds were normal, the abdomen was flat and soft, the liver and spleen were not touched under the ribs, and the systema nervosum was not abnormal. Chest radiograph and electrocardiogram were normal.

Blood test showed that leukocytes count was 11.15 × 10^9^/L (49% neutrophils; 34% lymphocytes), platelet count was 195 × 10^9^/L, C-reactive protein was 42.88 mg/L, Hypersensitive C reactive protein>50 mg/L, erythrocyte sedimentation rate was 69 mm/h, procalcitonin was 0.25ng/mL, liver and kidney function, myocardial enzyme, electrolyte were normal. Both EBV-DNA and antibody series were negative. *Mycoplasma pneumoniae*, *Chlamydia pneumoniae* antibody and tuberculosis antibody were negative. Respiratory syncytial virus, adenovirus, and parainfluenza virus antigen were negative. Blood culture was negative. Further examination of influenza virus nucleic acid for nasopharyngeal secretions was positive, and the influenza virus confirmed as influenza A (H1N1) pdm09 virus. B lymphocyte ratio (CD19+/CD45+) was 26%, B lymphocyte count (CD19+) was 840 cells/μL, Th/Ts ratio (CD4+/CD8+) was 3.79, and the counts and proportions of T lymphocytes and NK cells were normal.

Admission diagnosis was “influenza, acute laryngitis and second-degree larynx obstruction”. After admission, he received treatments including paramivir, latamoxef, dexamethasone, oxygen therapy, and so on. However, his fever was not relieved after the treatments. On the seventh day of the fever onset, B-ultrasound showed that the inner diameter of the left main coronary artery was dilated, and the double coronary artery was not smooth. After consultation with experts in KD, he was diagnosed as IKD and received treatments including human immunoglobulin (2 g/kg), aspirin (30∼50 mg/kg.d) and dipyridamole (3∼5 mg/kg.d). After 24 hours of human immunoglobulin infusion, his body temperature returned normal. After hospitalization for 6 days, his symptoms disappeared and discharged from the hospital. He continued to receive oral aspirin (10–15 mg/kg.d) treatment after discharge. The fiftieth days after the onset of the disease, the coronary artery was checked by echocardiography, and he was well-recovered. The results of disease course and the related inflammatory markers for this child were shown in Table 1.

**Table 1:**
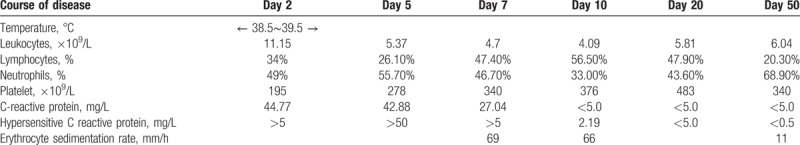
Results of disease course and the related inflammatory markers.

## 3 Discussion

It has been more than half a century since the discovery of KD; however, the etiology of KD is still unclear. Recently, the association between KD and pathogen infection has attracted more and more attention. Epidemiological surveys showed that the incidence of KD is high in winter and spring, which similar to many respiratory diseases. This seasonal feature suggests that KD may be caused by 1 or more pathogens associated with respiratory diseases.^[3]^

Previously, Joshi et al reported a case of new H1N1 infection accompanied by the occurrence of typical KD.^[4]^ Jeong et al and Shimada et al respectively, reported a typical KD in an 18-month-old boy and a 2-year-old girl after influenza vaccination with symptoms including fever, neck lymph node enlargement, conjunctival congestion, chapped lips, rash, fingertip swelling.^[5,6]^ These cases suggest that influenza virus may be closely related to the occurrence of KD. However, influenza virus infection associated with IKD has not been reported in the literature. Our case may be a useful supplement to the correlation between KD and influenza virus. In our case, after the child was infected with the new H1N1 influenza virus, his fever was not relieved by antiviral treatment. Although only cervical lymphadenopathy and fingertip flushing were found, progressive elevated platelet and persistent erythrocyte sedimentation rate >40 mm/h, C-reactive protein >30 ms/L, and coronary artery lesion that revealed by B-mode ultrasound on the seventh day of heat stroke can be diagnosed as IKD. After the corresponding treatment for IKD, the patient was gradually recovered. After eliminating the possibility of other pathogenic infections, the new H1N1 influenza virus infection may be a pathogenic factor. The viral infection leads to inflammation and abnormal lymphocyte subsets which were closely related to the occurrence of vascular inflammation in KD.

In the research of the relationship between pathogens and vasculitis, an analysis of adverse reactions to inflammatory diseases after immunization of the 3 international databases EV, VAERS, and VigiBase from 2003 to 2014, allergic purpura, KD and rheumatic myalgia are the most common adverse reactions. These findings indicated that pathogens may induce vasculitis of body.^[7]^ Rowley et al found that the up-regulation of interferon-stimulated gene expression was observed in acute lung tissue of KD, which indicated the existence of cellular immune response after viral infection. They also observed that coronary artery inflammation of KD has the characteristics of antiviral immune response, including the activation of cytotoxic T lymphocytes and type I interferon-induced up-regulation of relative genes. All these evidence strongly supported the hypothesis that viral infection may be associated with KD.^[8–10]^

## 4 Conclusion

In summary, more attention should be paid to the correlation between KD and pathogen infection, especially the new influenza virus H1N1. The potential mechanism underlying viral infection-mediated KD is worthy of further investigation, which may provide scientific evidence for the pathogenesis of KD.

## Acknowledgments

We thank the doctors of the Department of Infectious Diseases of Xi’an Children's Hospital for their help in data collection.

## Author contributions

**Conceptualization:** Jun Wang, Hui-Ling Deng.

**Data curation:** Jun Wang, Fang Sun, Hui-Ling Deng, Rui-Qing Liu.

**Formal analysis:** Fang Sun, Rui-Qing Liu.

**Investigation:** Fang Sun.

**Writing – original draft:** Hui-Ling Deng.

**Writing – review & editing:** Hui-Ling Deng.

Jun Wang orcid: 0000-0001-9408-2235.
